# Navigating the future of bacterial molecular epidemiology

**DOI:** 10.1016/j.mib.2010.08.002

**Published:** 2010-10

**Authors:** Stephen Baker, William P Hanage, Kathryn E Holt

**Affiliations:** 1 Oxford University Clinical Research Unit, Wellcome Trust Major Overseas Programme, Hospital for Tropical Diseases, 190 Ben Ham Tu, Quan 5, Ho Chi Minh City, Viet Nam; 2 Imperial College Faculty of Medicine, Department of Infectious Disease Epidemiology, St Mary's Hospital, Norfolk Place, London W2 1PG, United Kingdom; 3 Department of Microbiology and Immunology, The University of Melbourne, Parkville, Victoria 3010, Australia

## Abstract

Technological advances in high-throughput genome sequencing have led to an enhanced appreciation of the genetic diversity found within populations of pathogenic bacteria. Methods based on single nucleotide polymorphisms (SNPs) and insertions or deletions (indels) build upon the framework established by multi-locus sequence typing (MLST) and permit a detailed, targeted analysis of variation within related organisms. Robust phylogenetics, when combined with epidemiologically informative data, can be applied to study ongoing temporal and geographical fluctuations in bacterial pathogens. As genome sequencing, SNP detection and geospatial information become more accessible these methods will continue to transform the way molecular epidemiology is used to study populations of bacterial pathogens.

## Introduction

In 1854, as a cholera epidemic ravaged London's Soho district, John Snow investigated the outbreak by locating the location of cholera cases on a street map. From the distribution of cases, and by questioning local residents, Snow concluded that the source of the outbreak was a public water pump. This was the first epidemiological investigation to make use of maps and Snow is hailed as having founded the science of epidemiology [1]. Now, our understanding of bacteriology and mapping have improved to the extent that we can characterise an isolate by genome sequence, and precisely locate it using global positioning (GPS) technology.

Combining genetic, phenotypic, spatial and temporal data allows a comprehensive view of the epidemiology of bacterial pathogens and their evolution, helping to explain how virulence and other phenotypic traits evolve in bacterial species over time [2,3]. Adaptation occurs via a number of processes, including mutations, and the movement of genes among distinct lineages through recombination [4]. Both mutation and recombination produce genomic diversity that can be used to discriminate between related organisms. Multiple techniques have been employed to assay genomic differences among different lineages or clones of the same species, such as pulsed field gel electrophoresis (PFGE) and multi-locus variable number tandem repeat (VNTR) analysis (MLVA). The majority of these methods suffer from issues of portability and a limited understanding of the processes through which variation arises. By contrast, DNA sequence-based techniques provide robust and portable differentiation within bacterial populations, and can be used to infer their phylogenetic history.

Multi-locus sequence typing (MLST) is a well-established method to study bacterial populations exhibiting sufficient nucleotide diversity in a small number of genomic loci [5]. Databases containing MLST and associated data from hundreds or thousands of isolates can be accessed via the internet (http://www.mlst.net/ and http://pubmlst.org/) [6]. While MLST has provided numerous insights into the epidemiology and population genetics of bacteria, technological advances in DNA sequencing (e.g. 454, Illumina/Solexa and ABI SOLiD platforms) allow the rapid sequencing of entire bacterial genomes [7]. As a result, sequence-based analysis of bacterial populations exhibiting levels of nucleotide diversity too low for MLST has become possible [8]. Tagged genomic libraries can be used to generate sequence data from multiple isolates in a single assay, providing sufficient information to discover single nucleotide polymorphisms (SNPs), small insertions or deletions (indels) and variation in gene content in multiple bacterial strains over a short time frame.

It is recognised that the distribution of some bacteria may be related to geographical patterns, such as climatic zones and movement of human populations [9–11]. However, the spatial distribution of genetic variants can shed light on pathogen evolution and transmission. This type of analysis is aided by GPS devices that can be used to record the coordinates of relevant locations. The spatial distribution of bacterial pathogens can be considered at a local level (e.g. streets, hospital wards), at regional level (cities, provinces) or even globally. In a hospital setting this may indicate nosocomial transmission, but in a community setting, the simultaneous appearance of an identical genotype in widely dispersed locations may be a warning of an imminent epidemic.

## The phylo-geographical distribution of *Salmonella* Typhi

Defining the population structure of *Salmonella* Typhi (*S*. Typhi), the causative agent of the human restricted disease typhoid fever [12], has been, historically, particularly challenging. Typhoid is common in parts of Asia, South America and Africa, particularly in densely populated areas with poor sanitation. *S*. Typhi is genetically monomorphic, rendering MLST largely uninformative [13]. However, an approach that studied variation at 200 loci identified sufficient SNPs to define a minimum spanning tree containing 80 distinct haplotypes [14]. This comprehensive study, consisting of data from 105 strains isolated over 84 years on three continents, identified remarkable homogeneity with only very limited phylo-geographic signal. Furthermore, there was evidence for the persistence of multiple haplotypes in a single country over decades, indicating a stable population rather than clonal replacement by successively better-adapted lineages. Yet, there was evidence of a recent clonal expansion of a specific haplotype (H58) in Asia. Molecular typing of Vietnamese *S*. Typhi isolates suggests replacement of sensitive isolates with those predominantly of H58 haplotype frequently associated with multiple drug resistance (MDR), microevolution and acquired resistance mutations within this emerging clone [15,16].

**Figure 1:**
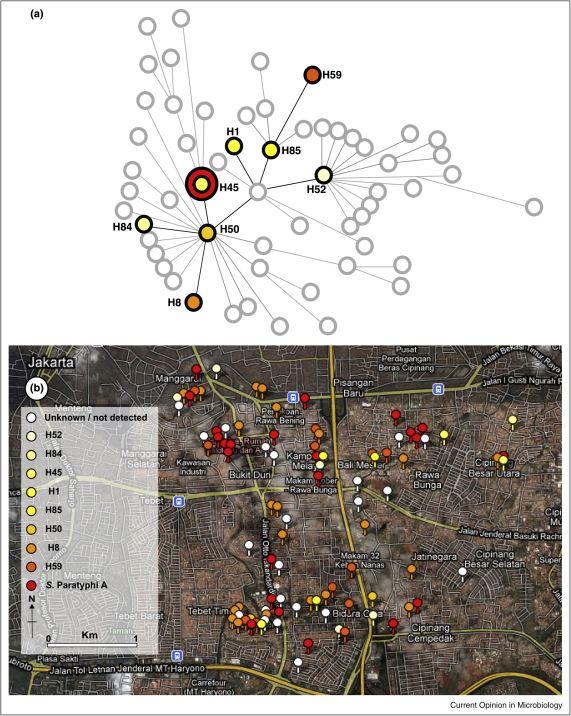
Google map and haplotype map outlining the circulation of multiple *Salmonella* Typhi haplotypes in a small urban area of Jakarta. The SNP typing of 54 *S*. Typhi strains from a single location in Jakarta identified several different haplotypes circulating within a two-year period [17]. **(a)** A minimum spanning tree showing relationships between the eight different *S*. Typhi haplotypes (e.g. H45) identified. The tree shows the overall population structure defined by the SNPs targeted in the assay, defined in ref. [14]. Coloured circles correspond to haplotypes found in the sample (colour corresponds to the colour scheme in part b below). Grey circles are haplotypes that were not identified among isolates from Jakarta. H45 is the ancestral group and the red circle denotes *Salmonella* Paratyphi A strains. **(b)** A Google maps image created by inputting data at http://www.spatialepidemiology.net/showing the local distribution of the various *S.* Typhi haplotypes and *S.* Paratyphi A in a suburb of Jakarta.

A high-throughput SNP detection platform was used to identify *S*. Typhi haplotypes circulating in an urban area of Jakarta [17] (Figure 1). The *S*. Typhi strains were isolated as part of a case/control study to identify risk factors for typhoid [18]. The SNP profiling of 140 *S*. Typhi strains identified nine haplotypes circulating in the Indonesian archipelago over more than 30 years, with eight detected in a single suburb over two years. One specific haplotype of *S*. Typhi was dominant and uniquely associated with an atypical flagella antigen [19]. These findings show that marked genotypic and phenotypic differences can exist within a relatively monomorphic pathogen population within a limited geographical area over a short time frame.

An additional ∼2000 SNPs have since been identified within the *S.* Typhi population, providing additional loci for more refined SNP typing of clinical isolates [20]. The sequencing of the whole genomes of 19 strains chosen to be representative of the global of *S*. Typhi, detected other forms of genetic variation (indels), which could, potentially, be used as markers for studying *S*. Typhi diversity. The development and use of a custom SNP array (containing over 1500 SNP loci) for *S*. Typhi using the GoldenGate platform (Illumina) provided greater discriminatory power than any previous study of *S*. Typhi. Application of this assay to *S*. Typhi populations in Nairobi, Kenya and Kathmandu, Nepal again showed multiple *S.* Typhi haplotypes co-circulating in a single city [21,22]. In both cities, however, a single haplotype was dominant, supporting the notion of clonal expansion rather than successive clonal replacement being the ongoing force in the population of this pathogen. Our current sequencing and SNP typing work relating specific haplotypes to the spatial and temporal distribution of typhoid cases in an area of Kathmandu, is expected to help elucidate specific transmission routes and microevolution within a highly localised area.

## The global dissemination of *Staphylococcus aureus*: unraveling tangled transmission routes

*S. aureus* is a major human pathogen in both hospital settings and the community, where it is a leading cause of skin and soft tissue infections. In most cases, *S. aureus* is carried asymptomatically by humans (and domestic animals, in which it can also be important pathogen in some contexts [23]). In healthcare settings the circulation of methicillin resistant strains (MRSA) is a constant challenge for infection control, and the emergence of MRSA as a cause of severe disease among healthy adults in the community is a cause for considerable concern [24].

**Figure 2:**
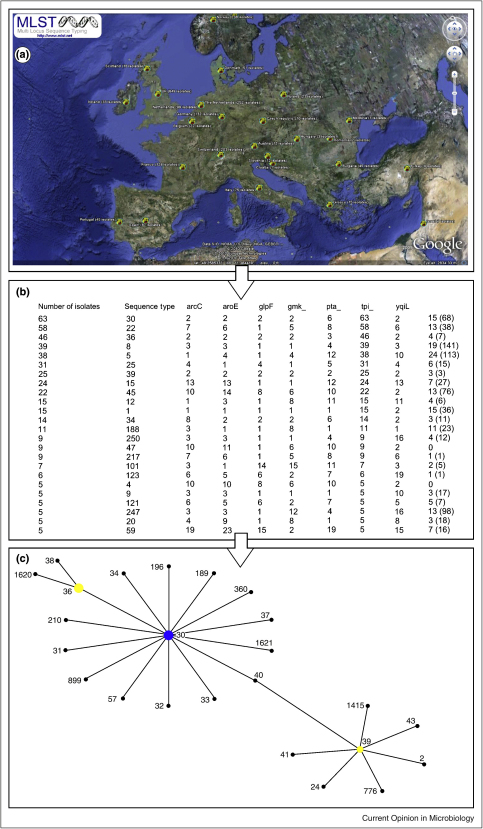
Combining and exploring MLST and geographical data for *Staphylococcus aureus* with MLST maps. MLST maps (http://maps.mlst.net/view_maps.php) allows the user to enter and integrate MLST data together with location as an additional variable. **(a)** Datasets can be downloaded (in this case *S. aureus*) and then opened and viewed in Google earth (http://earth.google.com/). The image shows the locations in Europe and the corresponding quantity of isolates of *S. aureus* with available MLST data. **(b)** By clicking on a country of origin the user can view the number of strains and the various sequences types (STs) in a text format. **(c)** MLST data for strains selected from within the MLST maps software can be sent directly to the clustering algorithm eBurst, allowing the user to identify groups of related genotypes in a single location or across multiple locations, in this case the *Staphylococcus aureus* ST30 clonal complex from the United Kingdom is shown.

The mainstays for studying the molecular epidemiology of *S. aureus* have been MLST [25] and staphylococcal protein A (*spa*) typing [26]. These two methods are augmented for MRSA with staphylococcal cassette chromosome (SCC*mec*, which carries the methicillin resistance gene, *mecA*) sequencing [27]. The MLST system has been enhanced by an interface with simple mapping data, where users can add and access geographical information (http://maps.mlst.net/view_maps.php) (Figure 2). The information gathered from MLST indicates that MRSA has evolved multiple times, leading to the circulation and predominance of particular clonal complexes and sequence types, for example ST5, ST225 and ST239 [28]. However, the repeated emergence of resistance on these backgrounds means that they are heterogeneous with respect to the type of *spa* gene and the *SCCmec* they carry.

Nübel *et al.* discovered 156 bi-allelic-polymorphisms (BiPs) in 138 global ST5 MRSA isolates [29]. These BiPs defined 89 haplotypes, which clustered according to the continent of isolation, but not the *spa* typing group. Furthermore, sublineages were found to be locally clustered. These data suggest that the global dissemination of MRSA is restricted and that locally dominant MRSA strains may be the result of *SCCmec* transfer into a strain of *S. aureus* that is pre-adapted, already exhibiting superior fitness.

A close relative of the well-described ST5 MRSA clone, namely ST225, has recently become increasingly prevalent in health care settings in central Europe [28]. The spatiotemporal dynamics of the spread of ST225 has been studied via mutation detection at 269 loci in a collection of 73 ST225 strains from Europe and the United States [30]. The ST225 MRSA strains demonstrated remarkable uniformity, with only 36 haplotypes (resulting from 48 BiPs) identified. This lack of diversity implied a recent common ancestor. A reconstructed ancestral scenario suggested the spread of this strain from Germany across central Europe, with the eventual expansion of the dominant clade from 1995 onwards [30]. This work illustrates the potential of combined sequence and spatial analysis to reconstruct strain dissemination events in the recent past.

ST239 is another widely dispersed lineage of MRSA, common in mainland Asia, South America and parts of Eastern Europe [31,32]. The genomes of 63 globally distributed ST239 isolates were recently sequenced using multiplex Illumina/Solexa sequencing [33]. SNPs identified among the 63 genomes revealed a strong phylo-geographic signal, with highly similar sequences identified in the same geographic area. A close relationship was noted between strains from Portugal and South America, which is suggestive of the historical and modern links between these two regions. In some cases, strains did not cluster by geography, and were considered to represent intercontinental transfer, including evidence of a single transmission event from Southeast Asia initiating an outbreak in the United Kingdom. This study also gave an indication that such a method may be suitable to study local transmission events. Five strains isolated over 13 weeks with a potential link from the same Thai hospital could only be differentiated by 14 individual nucleotide changes.

A glimpse of the future of bacterial molecular epidemiology may be offered by a recent study of the geographic distribution of differing MSSA and MRSA clones in Europe [34]. This work integrated data from 450 hospitals spanning 26 European countries and provided a snapshot of the current *S. aureus* strains circulating across Europe, identifying dominant *spa* types that form distinct geographical groups when compared using spatial statistics. Additionally, it introduced a public Web-based mapping and genotyping tool that could be applied to other organisms (http://www.spatialepidemiology.net/) (Figure 2). This online tool has also been integrated with a smartphone application (EpiCollect), making the collection and interrogation of epidemiological data in the field an existing reality [35]. The coordination of such a large network will act as a blueprint for conducting similar investigations and outlines an obvious direction for microbiological reference laboratory networks and surveillance systems.

## Conclusions

We have described several examples of recent work showing the potential of high-resolution genome sequencing for the study of the evolution of bacterial pathogens. This work has been enhanced by combining genomic data with epidemiological and geographical information. A general observation is that additional metadata (such as, disease syndrome, antimicrobial resistance phenotype and isolation date) are an increasingly important element in molecular based projects.

The examples we have considered are drawn from relatively clonal pathogens, meaning that recombination rates within these species are low. This is an important caveat as recombination can obscure phylogenetic signal, and so methods that rely on a robust phylogeny may be compromised. Recombination also has the potential to introduce phenotypic traits into different genetic backgrounds (e.g. SCC*mec* elements). One of the interesting questions that remains is the role of local clonal expansion, and the extent to which this is eroded (or perhaps facilitated) by the widespread movement of selected genes among lineages.

An understanding of the dynamics of bacterial populations can help to determine appropriate interventions, including, the use of vaccines, therapeutics, public health measures and ongoing pathogen surveillance. The combination of new technologies to gain increasingly accurate, high-resolution spatial and genetic data related to large populations of bacteria, promises to extend our understanding of the dynamics and transmission of pathogenic bacteria even further.

## Competing interests

The authors wish to declare that they have no competing interests.

## References and recommended reading

Papers of particular interest, published within the annual period of review, have been highlighted as:

• of special interest
•• of outstanding interest

## References

[1] Lilienfeld A.M., Lilienfeld D.E. (1984). John Snow, the Broad Street pump and modern epidemiology. Int J Epidemiol.

[2] Holden M.T., Feil E.J., Lindsay J.A., Peacock S.J., Day N.P., Enright M.C., Foster T.J., Moore C.E., Hurst L., Atkin R. (2004). Complete genomes of two clinical *Staphylococcus aureus* strains: evidence for the rapid evolution of virulence and drug resistance. Proc Natl Acad Sci USA.

[3] Baker S., Dougan G. (2007). The genome of Salmonella enterica serovar Typhi. Clin Infect Dis.

[4] Hanage W.P., Fraser C., Spratt B.G. (2006). The impact of homologous recombination on the generation of diversity in bacteria. J Theor Biol.

[5] Maiden M.C., Bygraves J.A., Feil E., Morelli G., Russell J.E., Urwin R., Zhang Q., Zhou J., Zurth K., Caugant D.A. (1998). Multilocus sequence typing: a portable approach to the identification of clones within populations of pathogenic microorganisms. Proc Natl Acad Sci USA.

[6] Aanensen D.M., Spratt B.G. (2005). The multilocus sequence typing network: mlst.net. Nucleic Acids Res.

[7] MacLean D., Jones J.D., Studholme D.J. (2009). Application of ‘next-generation’ sequencing technologies to microbial genetics. Nat Rev Microbiol.

[8] Achtman M. (2008). Evolution, population structure, and phylogeography of genetically monomorphic bacterial pathogens. Annu Rev Microbiol.

[9] Leimkugel J., Hodgson A., Forgor A.A., Pfluger V., Dangy J.P., Smith T., Achtman M., Gagneux S., Pluschke G. (2007). Clonal waves of Neisseria colonisation and disease in the African meningitis belt: eight-year longitudinal study in northern Ghana. PLoS Med.

[10] Suerbaum S., Achtman M. (2004). Helicobacter pylori: recombination, population structure and human migrations. Int J Med Microbiol.

[11] Vesaratchavest M., Tumapa S., Day N.P., Wuthiekanun V., Chierakul W., Holden M.T., White N.J., Currie B.J., Spratt B.G., Feil E.J. (2006). Nonrandom distribution of Burkholderia pseudomallei clones in relation to geographical location and virulence. J Clin Microbiol.

[12] Parry C.M., Hien T.T., Dougan G., White N.J., Farrar J.J. (2002). Typhoid fever. N Engl J Med.

[13] Kidgell C., Reichard U., Wain J., Linz B., Torpdahl M., Dougan G., Achtman M. (2002). Salmonella typhi, the causative agent of typhoid fever, is approximately 50,000 years old. Infect Genet Evol.

[14] Roumagnac P., Weill F.X., Dolecek C., Baker S., Brisse S., Chinh N.T., Le T.A., Acosta C.J., Farrar J., Dougan G. (2006). Evolutionary history of Salmonella typhi. Science.

[15] Weill F.X., Tran H.H., Roumagnac P., Fabre L., Minh N.B., Stavnes T.L., Lassen J., Bjune G., Grimont P.A., Guerin P.J. (2007). Clonal reconquest of antibiotic-susceptible Salmonella enterica serotype Typhi in Son La Province, Vietnam. Am J Trop Med Hyg.

[16] Le T.A., Fabre L., Roumagnac P., Grimont P.A., Scavizzi M.R., Weill F.X. (2007). Clonal expansion and microevolution of quinolone-resistant Salmonella enterica serotype typhi in Vietnam from 1996 to 2004. J Clin Microbiol.

[17] Baker S., Holt K., van de Vosse E., Roumagnac P., Whitehead S., King E., Ewels P., Keniry A., Weill F.X., Lightfoot D. (2008). High-throughput genotyping of Salmonella enterica serovar Typhi allowing geographical assignment of haplotypes and pathotypes within an urban District of Jakarta, Indonesia. J Clin Microbiol.

[18] Vollaard A.M., Ali S., van Asten H.A., Widjaja S., Visser L.G., Surjadi C., van Dissel J.T. (2004). Risk factors for typhoid and paratyphoid fever in Jakarta, Indonesia. JAMA.

[19] Baker S., Holt K., Whitehead S., Goodhead I., Perkins T., Stocker B., Hardy J., Dougan G. (2007). A linear plasmid truncation induces unidirectional flagellar phase change in H:z66 positive Salmonella Typhi. Mol Microbiol.

[20] Holt K.E., Parkhill J., Mazzoni C.J., Roumagnac P., Weill F.X., Goodhead I., Rance R., Baker S., Maskell D.J., Wain J. (2008). High-throughput sequencing provides insights into genome variation and evolution in Salmonella Typhi. Nat Genet.

[21] Holt K., Baker S., Dongol S., Basnyat B., Adhikari N., Thorson S., Pulickal A., Song Y., Parkhill J., Farrar J. (2010). High-throughput bacterial SNP typing identifies distinct clusters of *Salmonella* Typhi causing typhoid in Nepalese children. BMC Infect Dis.

[22] Kariuki S., Revathi G., Kiiru J., Mengo D.M., Mwituria J., Muyodi J., Munyalo A., Teo Y.Y., Holt K.E., Kingsley R.A. (2010). Typhoid in Kenya is associated with a dominant multidrug resistant Salmonella Typhi haplotype that is also widespread in South East Asia. J Clin Microbiol.

[23] Morgan M. (2008). Methicillin-resistant *Staphylococcus aureus* and animals: zoonosis or humanosis?. J Antimicrob Chemother.

[24] Klein E., Smith D.L., Laxminarayan R. (2007). Hospitalizations and deaths caused by methicillin-resistant *Staphylococcus aureus*, United States, 1999–2005. Emerg Infect Dis.

[25] Enright M.C., Day N.P., Davies C.E., Peacock S.J., Spratt B.G. (2000). Multilocus sequence typing for characterization of methicillin-resistant and methicillin-susceptible clones of *Staphylococcus aureus*. J Clin Microbiol.

[26] Harmsen D., Claus H., Witte W., Rothganger J., Claus H., Turnwald D., Vogel U. (2003). Typing of methicillin-resistant *Staphylococcus aureus* in a university hospital setting by using novel software for spa repeat determination and database management. J Clin Microbiol.

[27] Katayama Y., Ito T., Hiramatsu K. (2000). A new class of genetic element, staphylococcus cassette chromosome mec, encodes methicillin resistance in *Staphylococcus aureus*. Antimicrob Agents Chemother.

[28] Enright M.C., Robinson D.A., Randle G., Feil E.J., Grundmann H., Spratt B.G. (2002). The evolutionary history of methicillin-resistant *Staphylococcus aureus* (MRSA). Proc Natl Acad Sci USA.

[29] Nubel U., Roumagnac P., Feldkamp M., Song J.H., Ko K.S., Huang Y.C., Coombs G., Ip M., Westh H., Skov R. (2008). Frequent emergence and limited geographic dispersal of methicillin-resistant *Staphylococcus aureus*. Proc Natl Acad Sci USA.

[30] Nubel U., Dordel J., Kurt K., Strommenger B., Westh H., Shukla S.K., Zemlickova H., Leblois R., Wirth T., Jombart T. (2010). A timescale for evolution, population expansion, and spatial spread of an emerging clone of methicillin-resistant *Staphylococcus aureus*. PLoS Pathog.

[31] Bartels M.D., Nanuashvili A., Boye K., Rohde S.M., Jashiashvili N., Faria N.A., Kereselidze M., Kharebava S., Westh H. (2008). Methicillin-resistant *Staphylococcus aureus* in hospitals in Tbilisi, the Republic of Georgia, are variants of the Brazilian clone. Eur J Clin Microbiol Infect Dis.

[32] Feil E.J., Nickerson E.K., Chantratita N., Wuthiekanun V., Srisomang P., Cousins R., Pan W., Zhang G., Xu B., Day N.P. (2008). Rapid detection of the pandemic methicillin-resistant *Staphylococcus aureus* clone ST 239, a dominant strain in Asian hospitals. J Clin Microbiol.

[33] Harris S.R., Feil E.J., Holden M.T., Quail M.A., Nickerson E.K., Chantratita N., Gardete S., Tavares A., Day N., Lindsay J.A. (2010). Evolution of MRSA during hospital transmission and intercontinental spread. Science.

[34] Grundmann H., Aanensen D.M., van den Wijngaard C.C., Spratt B.G., Harmsen D., Friedrich A.W. (2010). Geographic distribution of *Staphylococcus aureus* causing invasive infections in Europe: a molecular-epidemiological analysis. PLoS Med.

[35] Aanensen D.M., Huntley D.M., Feil E.J., al-Own F., Spratt B.G. (2009). EpiCollect: linking smartphones to web applications for epidemiology, ecology and community data collection. PLoS One.

